# Drs. Taft & Watt

**Published:** 1854-01

**Authors:** 


### DRS. TAFT & WATT.

In our notice of the Crystal Gold preparation of these gentlemen, we omitted to say that so soon as they complete the experiments they are making for the perfection of this article, they intend giving the Profession the benefit of their experience in the mode of preparing it. In the meantime, they are supplying the Profession as fast as they can, and are anxious that all may test it most thoroughly.

